# High performance microservice communication technology based on modified remote procedure call

**DOI:** 10.1038/s41598-023-39355-4

**Published:** 2023-07-26

**Authors:** Lei Zhang, Ke Pang, Jiangtao Xu, Bingxin Niu

**Affiliations:** 1 School of Microelectronics, Tianjin University, Road No 92, Tianjin Wei Jin Road, Nankai District City, Tianjin, 300072 China; 2 School of Software and Communication, Tianjin Sino-German University of Applied Sciences, Tianjin, 300350 China; 3 School of Artificial Intelligence, Hebei University of Technology, Hebei, 300401 China

**Keywords:** Computer science, Software

## Abstract

Microservice architecture is a programming method that decomposes a single application into various smaller services and then executes them. However, this approach introduces new challenges in communication between services because of the different data structures and technology types among the multiple services. Therefore, interprocess communication (IPC) between services has become one of the important challenges facing microservice architecture. Additionally, the choice of IPC technology is an important decision that can affect the nonfunctional requirements of the entire architecture. To address this problem, this study proposes a microservice communication technology based on remote procedure calls (RPC) called RPCX to improve the communication performance between services. The RPCX communication mechanism based on RPC uses the nonblocking IO communication model and Protobuf data serialization standard method. It identifies RPC communication at the client and server ends using dynamic proxy and annotation configuration technology. We use RPCX and two traditional service communication technologies to conduct performance stress benchmarking and evaluate the performance of RPCX through the time consumed to process the requests and transactions per second (TPS) performance stress indicators. The results show that the performance of RPCX is better than that of the other two technologies under different threads and requests. In this study, we show that RPCX has overall better performance than the other two service communication techniques under different threads and requests.

## Introduction

Microservice architecture is a programming method that decomposes a single application into various smaller services and then executes them^1^. In view of the shortcomings of large-scale monolithic applications in terms of maintenance, reusability, and expandability^2^, the microservice architecture divides the applications into multiple services for development, which are easier to maintain and expand. Based on this architecture, developers can choose the most suitable technology type for each service, which reduces the development cost and improves the development efficiency^3–5^. Nevertheless, this approach introduces new challenges in communication between services because of the different data structures and technology types among the multiple services. Therefore, interprocess communication (IPC)^6^ between services has become one of the important challenges facing microservice architecture^7^.

In the development of microservice architecture, the choice of IPC technology is an important decision that can affect the nonfunctional requirements of the entire architecture^7,8^. The commonly used IPC technology is obtained by remote procedure call (RPC)^9^ or representational state transfer (REST) technology^10–12^. Therefore, the selection of these two types of communication technologies is crucial in the service communication development of microservices.

We propose a microservice communication technology called RPCX, which adopts RPC and uses the nonblocking IO (NIO) communication model^13^, and Protobuf data serialization standard^14^ to establish a communication bridge between the client and server. It also uses dynamic proxy^15^ and annotation configuration technology^16^ to obtain dual-end RPC communication technology between the client and server. Thus, it renders RPC communication more efficient and convenient.

The contributions of this study include:

1. A microservice communication technology based on the RPC communication mechanism-RPCX. Compared with other technologies, this technology significantly reduces the service communication time and increases the transactions per second (TPS). The stress test under different threads and requests shows that the time-consuming performance is 55.9–88.9% higher than that of other technologies. The TPS is 126.9–802.8% higher than that of other technologies;
2. The user calls the remote service method locally without perception. To improve the ease of use, the dynamic agent technology and annotation configuration rules are introduced in RPCX, so that developers can use local methods to call remote service methods without knowing how the bottom layer operates, and achieve the purpose of two-stage service communication; and
3. Buffer pool technology. To further improve the performance of RPCX, time-consuming operations are input into the buffer pool at the program initialization stage for efficient data reading by the program at the running stage.

The rest of this paper is organized as follows. Section "Related work" introduces the existing literature in related fields. Section "RPCX" describes the design method of RPCX from four key aspects: dynamic proxy technology, annotation configuration rules, network communication model, and transmission data format. Section "Experiment" experimentally compares RPCX with two other communication technologies in terms of computational stress performance. Section "Conclusions" summarizes the findings and outlook.

## Related work

The performances of various IPC communication technologies and their impact on the overall performance of microservices were compared and analyzed in the extant literature. For example, Kumar et al.^2^, Shafabakhsh et al.^17^, and Hong et al.^18^ discussed and compared the performance indicators of various communication technologies, such as Google Remote Procedure Call (gRPC), Thrift, REST, and RabbitMQ, and proposed the best application scenarios for them. Gan and Delimitrou^19^ established a microservice system for streaming media services to evaluate various indicators in microservices, including the performance impact of RPC communication between microservices on the entire system. Georgiou and Spinellis^20^ discussed the energy consumption of various IPC communication technologies under different programming languages.

Gan et al.^21^, Sriraman and Wenisch^22^, Ueda et al.^23^, and others focused on the impact of communication technology on the performance of microservice architectures. Gan et al.^21^ also analyzed the time taken to process a communication request with respect to the time taken by the entire application. Sriraman and Wenisch^22^ developed a suite of microservices to analyze the influence of the operating system and communication requests on the overall latency of microservices. Ueda et al.^23^ tested the same on a monolithic application and a microservice architecture, respectively, and concluded that the optimization of communication between services can improve the overall performance.

To summarize, the performance of communication technology contributes to the overall performance of the microservice architecture. Therefore, we proposed an efficient microservice communication technology called RPCX, which uses the nonblocking IO network model and the Protobuf data transmission format as the underlying communication mechanism and uses dynamic proxy technology and annotation configuration rules to allow developers to call methods locally. The purpose of the remote method is to use the buffer pool technology to input time-consuming operations in the buffer pool for the program to read data at high speed during the running phase. It will play a positive role in promoting the development of the microservice IPC communication technology field.

## RPCX

First, the structure of the high-performance remote communication technology (RPCX) is described. Next, the four key components of the technology, namely dynamic proxy, annotation configuration rules, network communication model, and transmitted data format, are described.

### Overall structure of RPCX technology

Our objective for RPCX is to enable local applications to call services at remote servers with more ease and efficiency. Accordingly, we describe the four components mentioned in the previous paragraph: dynamic proxy, annotation configuration rules, network communication model, and transmitted data format.

**Figure 1:**
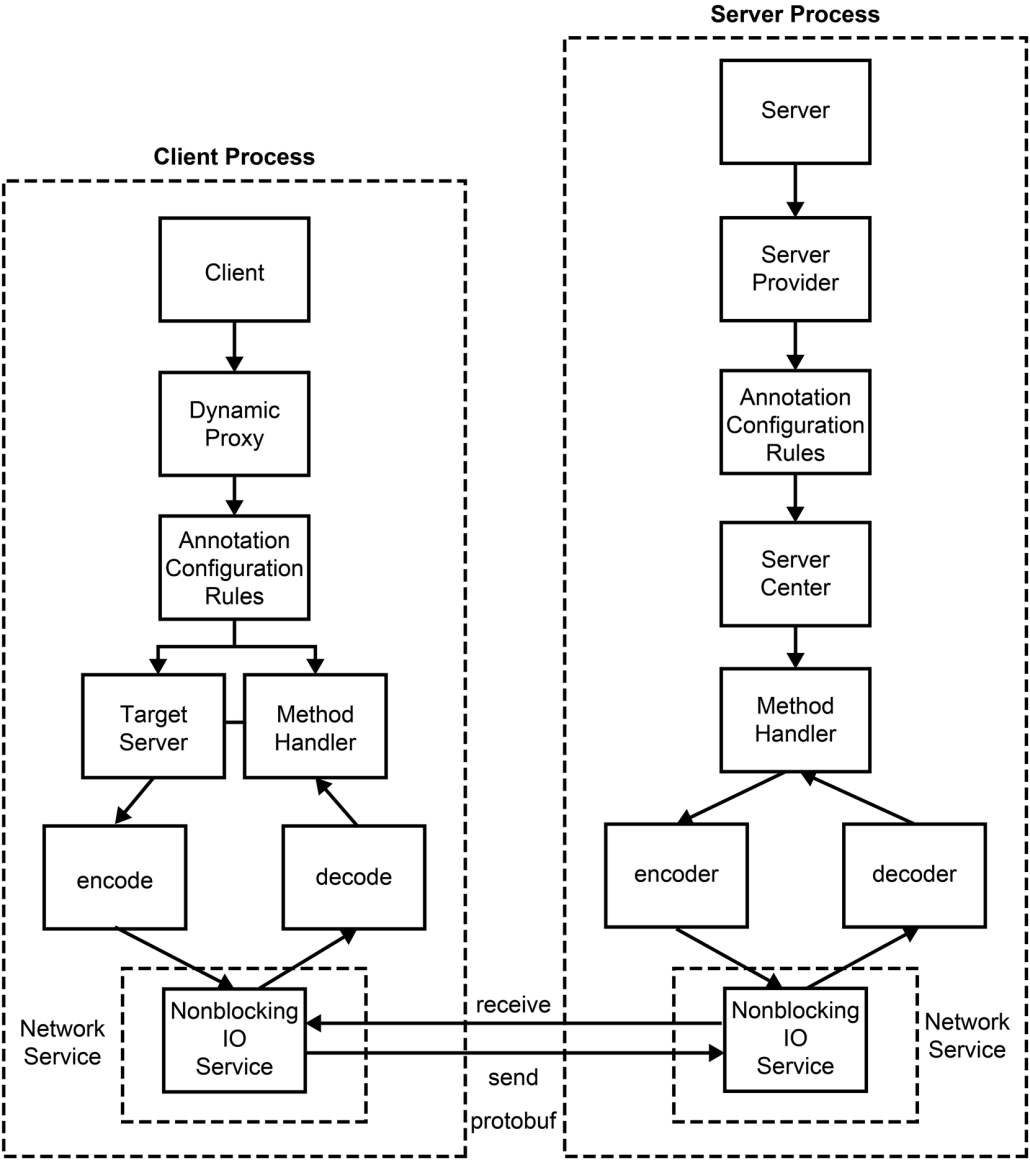
Overall structure of RPCX communication technology.

As illustrated in Fig. 1, RPCX is based on the principle of RPC remote communication. It is divided into two parts: client and server processes. In the client part, the remote proxy object is implemented first. RPCX used dynamic proxy technology to proxy the remote proxy service as a local service. The information required to locally call remote services, such as server IP information, service name, parameter names, and values, was locally annotated to disguise the service name and other information, obtain service positioning, and enable developers to implement it. Corresponding information could be directly obtained via the provided annotation class. The communication model was implemented using the nonblocking IO (NIO) communication model. The communication data were implemented using Protobuf. The required information was encoded into binary data, which were transmitted to the server through the network communication model service for decoding. The binary result data returned by the server were locally decoded and returned to the “method handler” for processing.

The server part was divided into service provider and service center. The service provider was used to provide the service interface definition and service implementation method. On the one hand, the service center was responsible for publishing the local service of the server as a remote service to provide services to the client. On the other hand, it managed remote services and could perform operations such as start, stop, and obtain the port number for the remote services. Developers could disguise service names through annotations and then publish the disguised service names for unified management at the service center. Once the binary data were received through the NIO network service on the server, the data were decoded to obtain the information required by the service. After the service was executed, the result was encoded, and the binary data of the result were returned to the client.

### Key components

#### Dynamic proxy

**Figure 2:**
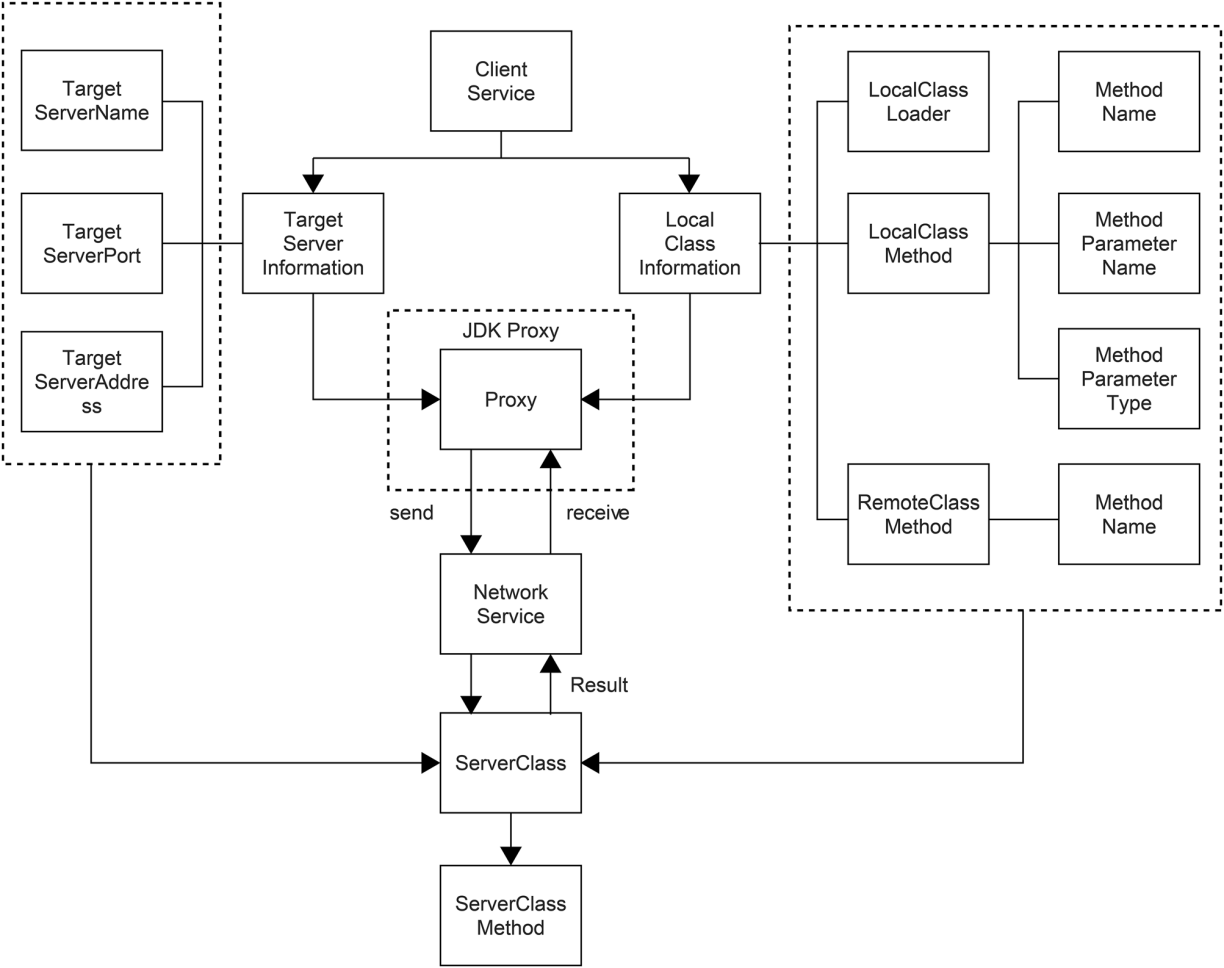
Dynamic proxy structure.

The purpose of a dynamic proxy is to call methods remotely on the server side, similar to methods on the client side, so that users can call remote methods locally without being aware of it. Therefore, while calling the local method, the user only requires the information of the remote server, namely the server address, port number, name, and the name of the server method. RPCX uses Java SE Development Kit (JDK) Proxy to create a proxy object, access the target server, and then transmit the remote method name, local method parameter value, and parameter type to the server. Upon receiving the method execution result from the server, it will return the value of the method, obtaining the effect of dynamically calling the local method to obtain the remote server method’s return value during the runtime of the program (Fig. 2).

#### Annotation configuration rules

To improve user friendliness and secure the application programming interface (API) exposed to the server, RPCX uses an annotation configuration rule to disguise the server information and configure it on the client side. For example, the @RPCXClient annotation class is used for the called local interface class. This annotation class has three attributes, RemoteServerName, RemoteServerDomain, and RemoteServerAddress, which represent the remote server service name, remote server port number, and remote server address, respectively. Through this annotation, the remote server information to be called can be directly marked on the local interface class, reducing the steps of feeding relevant information into the configuration file. In the local interface method of the client, @Method can be used to annotate the class to mark the name of the remote server method to be accessed on the local method. This enables the remote server method to be called locally.

When using the annotation class, the remote server can annotate the service and method names that it requires to publish through the @PRCXServer and @Method annotation classes. The published name might not be the same as the actual name; hence, RPCX takes the annotated name as the standard. Thus, the actual name is disguised, and the actual information is masked, enhancing the security of server-side information.

**Figure 3:**
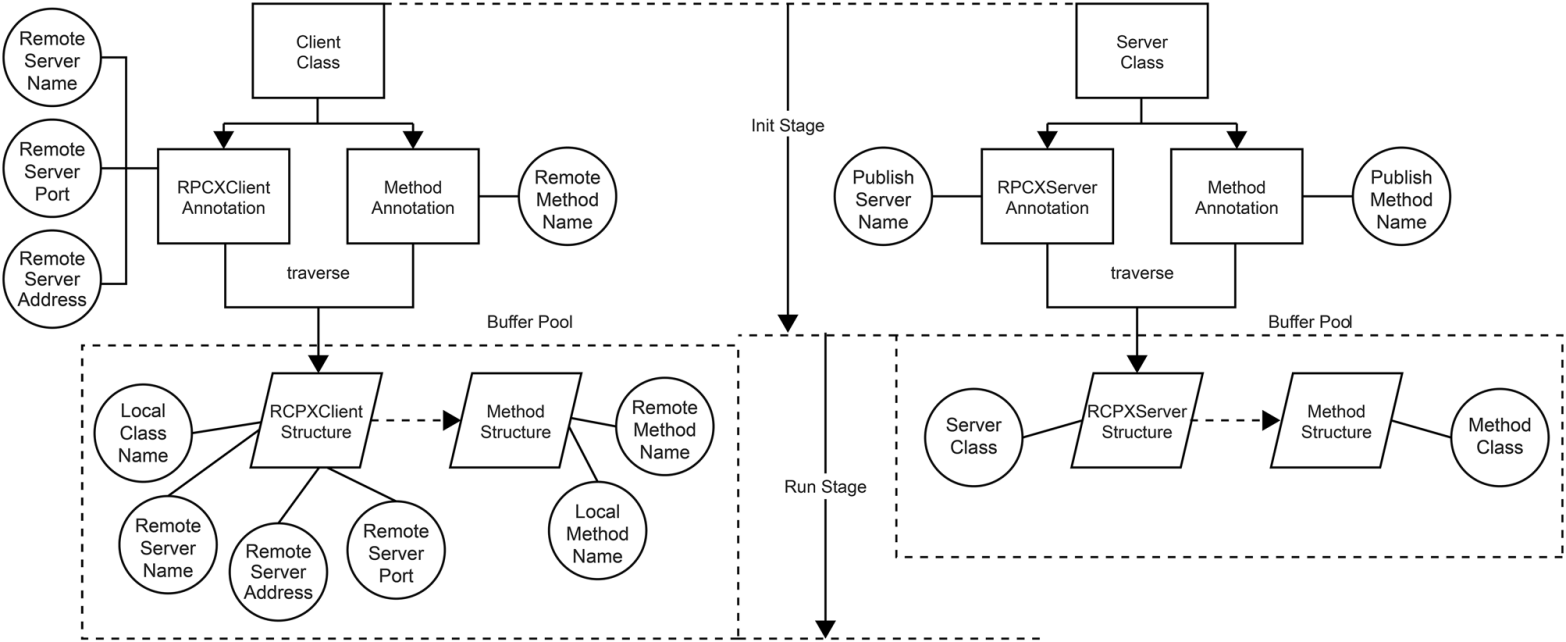
Annotation configuration design structure.

For better performance, in the initialization phase, RPCX recursively traverses all the annotation classes in this project and converts them into a unified structure. It can directly use this structure to obtain the local and remote server information during runtime (Fig. 3).

#### Network communication model

**Figure 4:**
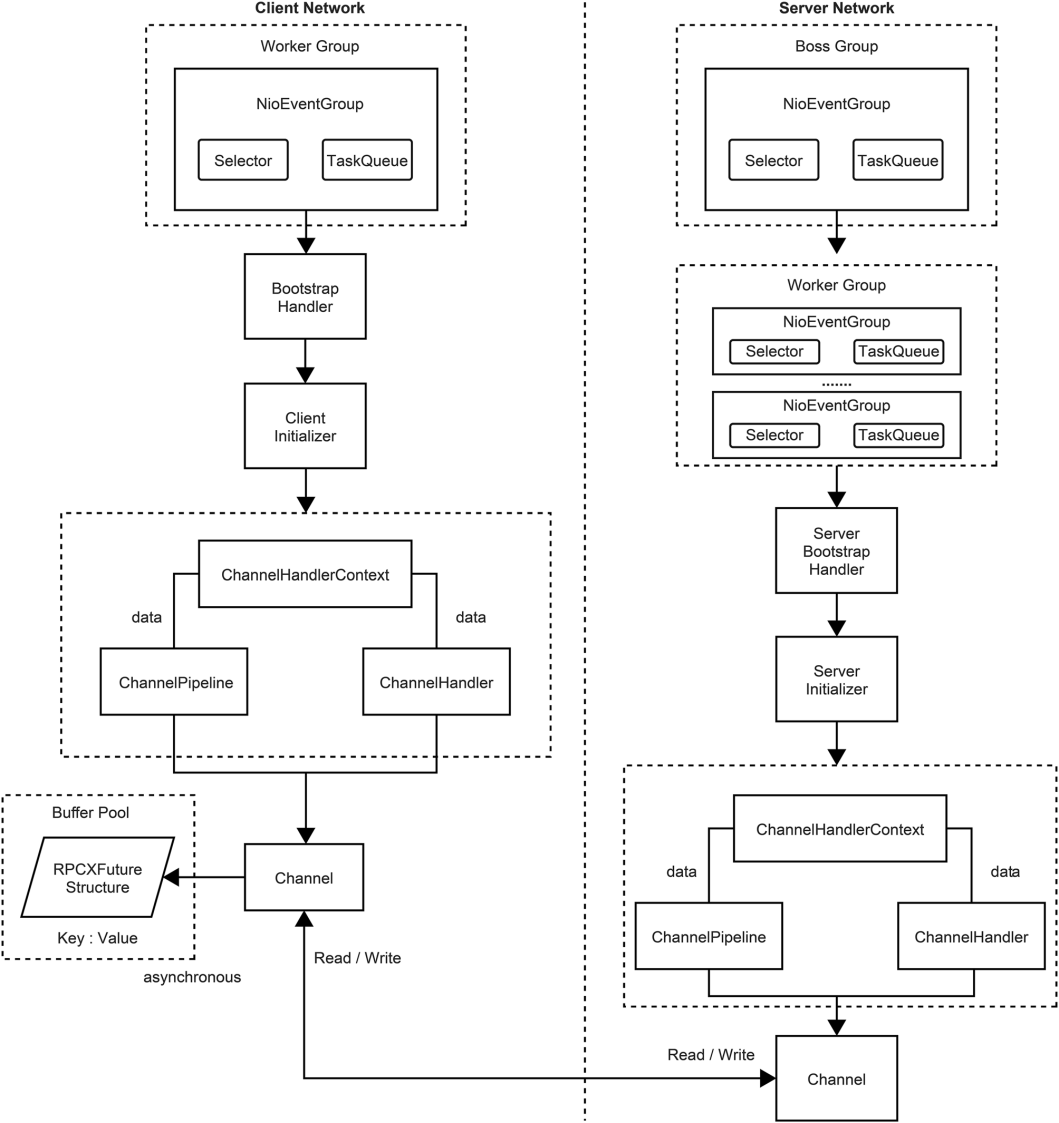
Network communication model structure.

The network communication model of RPCX adopted Netty’s asynchronous NIO design (Fig. 4). For this, we created NioEventLoop threads on the client and server sides, configured each component through Bootstrap and ServerBootstrap to start and guide, and executed data I/O operations through the channel pipeline.

The client transfers data to the server in binary format. Upon receiving the data, the server decodes and serializes it and then returns the result to the client in the same manner. However, because of the multithreaded asynchronous nature of Netty’s I/O operations, when the server returns the result, the identity of the thread that transferred the result is not revealed to the client. To solve this problem, when the client transfers data, the unique identifier of the request was added, and a key-value pair, with the key as the request identifier and the value as the RPCXFuture structure, was established in the client cache pool. RPCXFuture was used to save the serialized data returned by the server. This result would be stored in the key-value pair of the client’s cache pool with the request ID as the key, so that the client could receive a unique result corresponding to the request when calling.

#### Transmission data format

**Figure 5:**
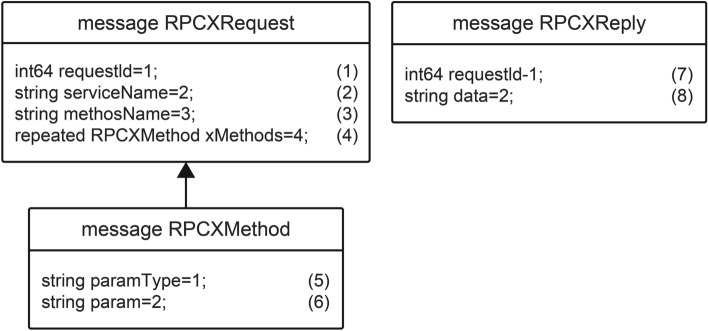
Protobuf transmission format design.

RPCX employs Protobuf as the transmission data’s format (Fig. 5). The client sends the RPCXRequest message body to the server, where (1) is the unique identifier of the request. As described in the network communication model, the identifier is used to indicate the uniqueness of the result of the transmission request when sending and receiving multithreaded messages. This identifier is a random long number. (2) and (3) are the service and method names sent to the server after information disguise, respectively. (4) Is a group of method array message bodies containing (5) method parameter types and (6) method parameter values. The RPCXReply message body is the data structure of the method returned by the server upon receiving the RPCXRequest request. The server adds the unique identifier (7) in the request and the result data (8) returned by the method into the RPCXReply and subsequently relays it to the client in the form of binary data. After the client receives the result returned by the method, the client structures data according to the return type of the method, which forms a complete data path for locally calling the remote method.

## Experiment

Here, we compared the proposed RPCX with two service communication technologies in terms of stress performance, collated the experimental results, and interpreted them.

### Experimental environment

#### Experimental platform

The experimental platform consists of two cloud servers with identical configurations. The specifications of each cloud server are as follows: 1 vCPU, 2 GB of memory, 40 GB of cloud storage, and 1 Mbps of bandwidth. The cloud servers are equipped with the CentOS 8.2 64-bit operating system, and the JDK 1.8 runtime environment has been installed.

#### Service communication technology

The communication technologies for the comparison were selected from REST and gRPC. REST is a stateless architectural style in distributed systems widely used to provide globally accessible APIs. In microservices, developers commonly use Spring’s OpenFeign, a REST technology^20^. OpenFeign is a declarative WebService client with the core function of providing simple and efficient RPC calls for the REST in the form of an HTTP method. gRPC is a cross-platform, open-source, and high-performance RPC framework developed by Google. It uses Protocol buffers 3 and http/2 to boost its speed and interoperability between services. We selected gRPC as several companies using microservices (e.g., Netflix, Cisco, Coreos) are adopting it in their production lifecycle^20,24^.

#### Experimental architecture

**Figure 6:**
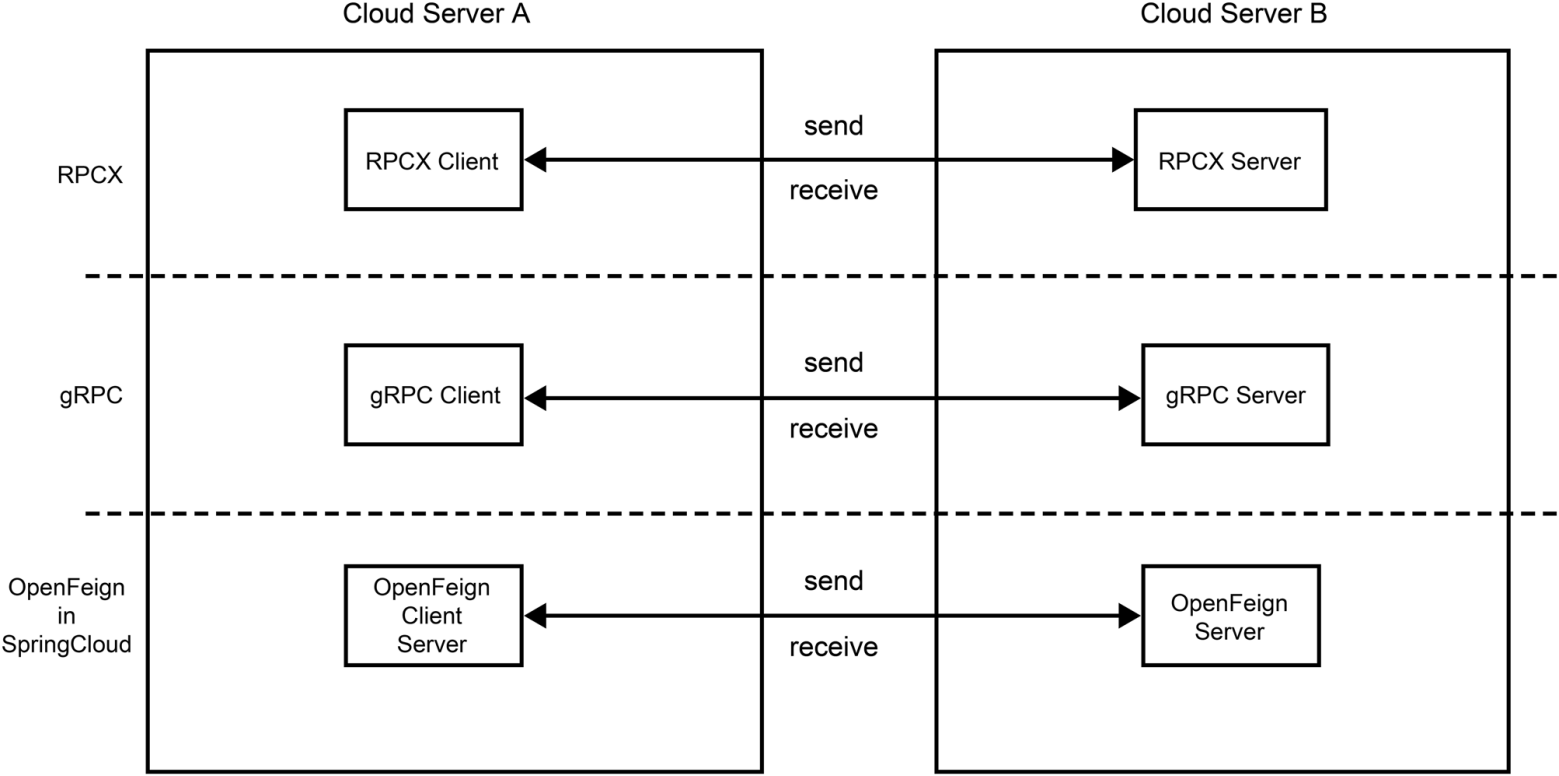
Experimental architecture.

In this experiment, we have set up two cloud servers with identical configurations: cloud server A and cloud server B. Cloud server A serves as the client-side server for RPCX, gRPC, and OpenFeign and is used to send information, whereas cloud server B serves as the server-side server and is used to receive information sent from cloud server A and return results. The performance testing experiment platform architecture for these three technologies is shown in Fig. 6.

To ensure the fairness of the experiment, the data transmitted by RPCX, gRPC, and OpenFeign in the experiment had the same strings. Both gRPC and OpenFeign used the example method given on the official website^24,25^. The key software versions used in the experiments are presented in the following table (Table 1).

**Table 1:** Software versions used for the RPCX versus benchmark stress performance test.

Communication technology	Technical name	Version	Link
RPCX	JDK	1.8	https://www.oracle.com/hk/java/technologies/javase/javase8u211-later-archive-downloads.html
	Netty	4.1.76	https://netty.io/downloads.html
	Protobuf	3.20.0	https://github.com/protocolbuffers/protobuf
gRPC OpenFeign	gRPC	1.45	https://github.com/grpc/grpc-java
	SpringBoot	2.7.11	https://spring.io/projects/spring-boot
	SpringCloud	2021.0.7	https://spring.io/projects/spring-cloud
	Openfeign	3.1.7	https://spring.io/projects/spring-cloud-openfeign

*RPCX* remote communication technology, *JDK* Java SE Development Kit, *gRPC* Google Remote Procedure Call.

### Performance experiment method

To evaluate the performance of the RPCX communication technology, we conducted a performance stress benchmark test by comparing it with gRPC and OpenFeign technologies. The stress benchmark test is a method of evaluating the performance of related technologies by simulating multi-threaded and multi-request scenarios to test program runtime and transactions per second (TPS). To test these two indicators, we developed a client-side testing program. The pseudocode for the testing program is as follows:

The pseudocode for the client-side testing program
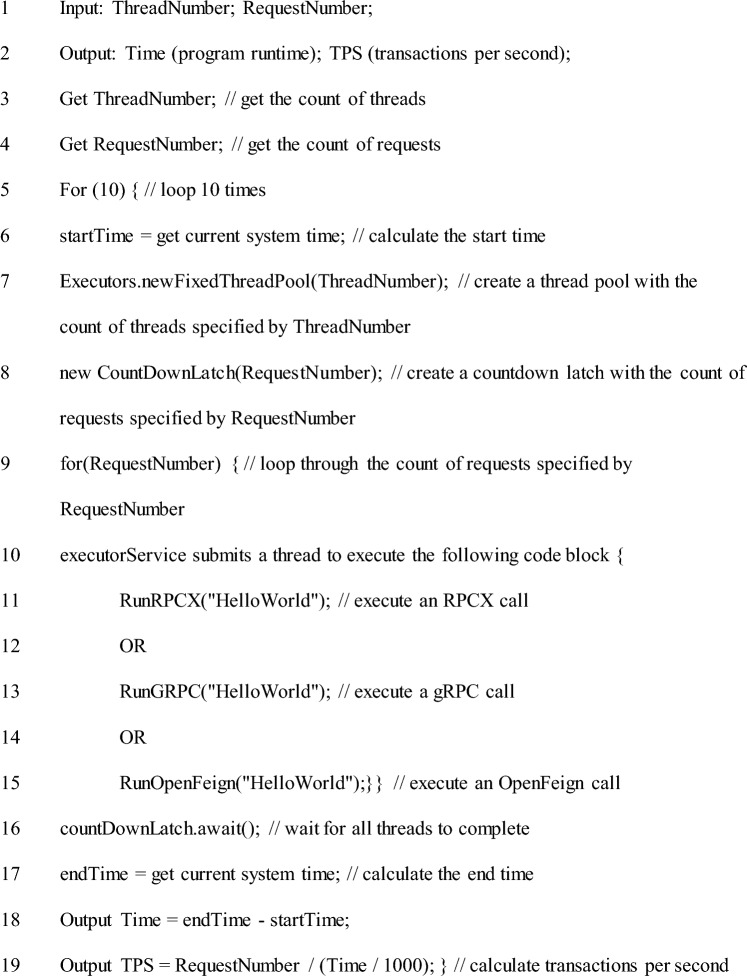


The loop operation in line 5 of the client-side testing program is designed to run each communication technology 10 times under the same count of threads and requests and record the consumption time of each run. The average program runtime and TPS of the 10 runs are then calculated. The purpose of this operation is to test the performance of each communication technology in a fair and accurate manner and to avoid the impact of exceptional conditions, such as server or communication failures, on the experimental data of a single run, which may affect the overall accuracy and reliability of the experimental results.

The test program takes thread count and request count as input variables. In Eqs. (1) and (2), we define the thresholds for thread count and request count, where α and β are integers.

1 $$\alpha \le TheadNumber\le \beta$$

2 $$\alpha \le RequestNumber\le \beta$$

As shown in line 19 of the testing program, TPS is an important metric for measuring the processing capability of a system in stress performance testing. In this experiment, it can be calculated from the count of requests and program runtime as follows:

3 $$TPS=\frac{\mathrm{RequestNumber}}{\mathrm{Time}/1000}$$

In Eq. (3), the program runtime (expressed as time) is in milliseconds; hence, when calculating the TPS, we divided the request by 1000 to convert the value to seconds.

The performance stress tests of the three communication technologies simulate data communication between the client-side and the standardized server-side program under different thread and request counts. Therefore, the server-side testing program serves to start and interact with the client-side for data communication. As a result, the server-side testing programs for gRPC and OpenFeign are examples of the official startup of the server-side program. In contrast, the server-side testing program for RPCX has the structure shown in Section "Overall structure of RPCX technology", with pseudocode as follows:

Pseudocode of Server-side Test Program.
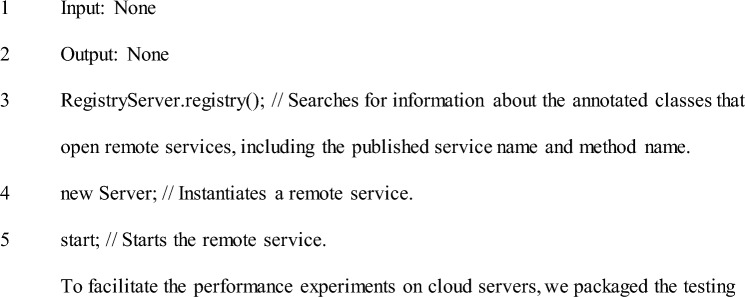


To facilitate the performance experiments on cloud servers, we packaged the testing programs of RPCX, gRPC, and OpenFeign into component packages with a file extension of .jar, which we refer to as jar packages. The jar packages of the three communication technologies are divided into client-side and server-side, and uploaded to cloud servers A and B, respectively, with irrelevant threads closed to maximize the utilization of server resources for testing program execution.

To maximize the stress performance of RPCX, gRPC, and OpenFeign communication technologies on cloud servers, a technical performance testing plan was developed that covers the full range of the two input parameters of the testing program: thread count and request count. That is, the thread count and request count start at 10 and continue to accumulate until their values are large enough to exhaust server resources, resulting in an infinitely prolonged communication time that makes communication impossible. Based on this strategy, the threshold of α and β in Eqs. (1) and (2) is from 10 to + ∞. Under the same thread count, the request count will run from 10 to + ∞ once in a loop, with both the thread count and request count increasing by 10 each time. To implement this strategy, we wrote 115 lines of shell commands to loop and run the testing program on the cloud server, with pseudocode as follows:

Pseudocode for running a test program in the shell;
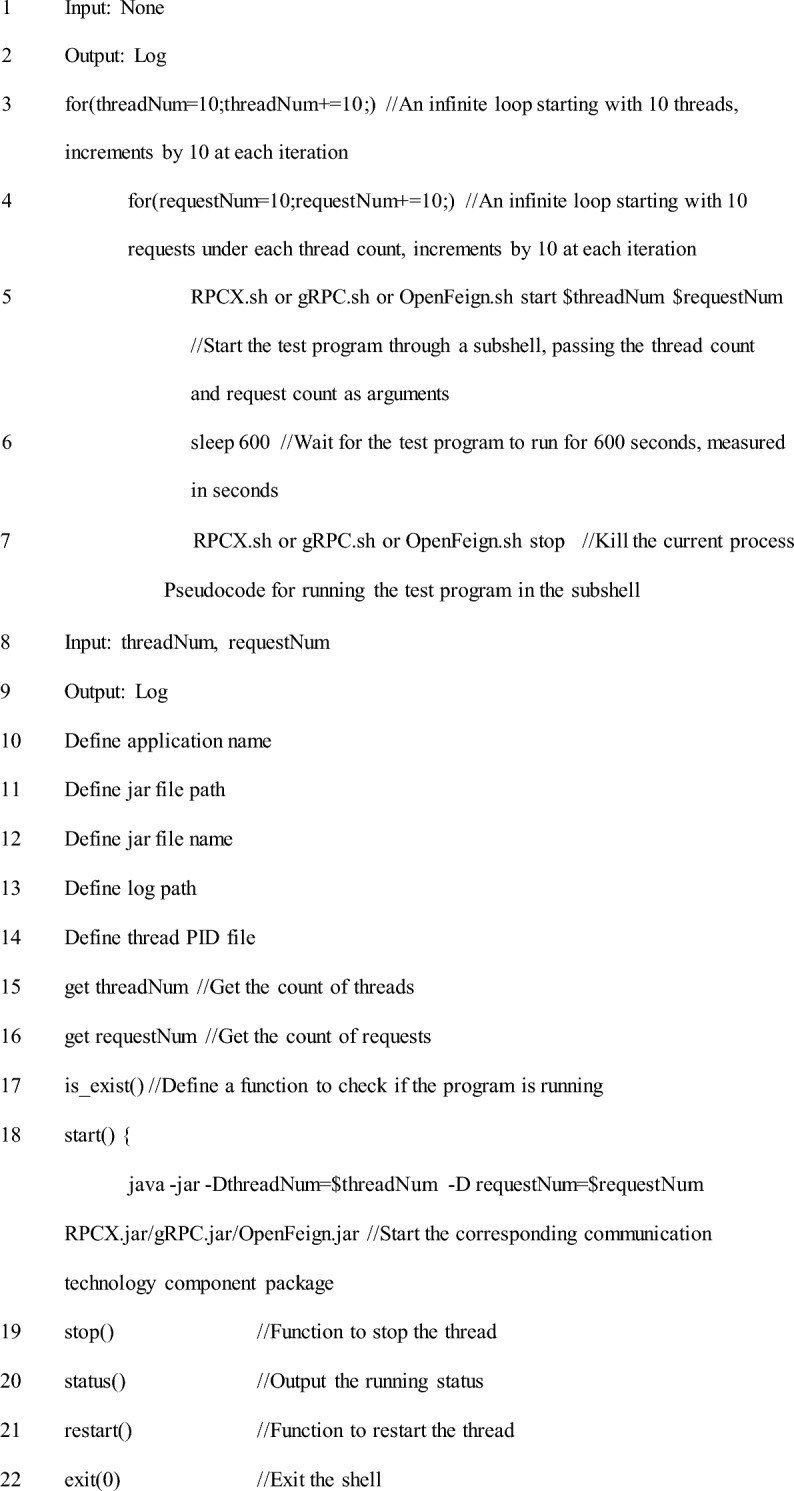


In our experiments, we found that when server resources are exhausted and communication cannot be established, the runtime of a single-cycle program is generally not more than 10 min. Additionally, communication exceeding 10 min is often meaningless in practical applications. Therefore, we set the waiting time for program execution on line 6 of the test shell to 600 s. We consider communication to have failed if the single-cycle communication time exceeds 10 min, and the communication result cannot be waited for.

### Experimental data analysis

During the experiment, to save server resources used for data storage, the performance stress test results of the three communication technologies were stored in real-time on the server in the form of files with thread-request count units, totaling 186,860 data. After the experiment, we needed to store the experimental data in a database for further data analysis.

**Figure 7:**
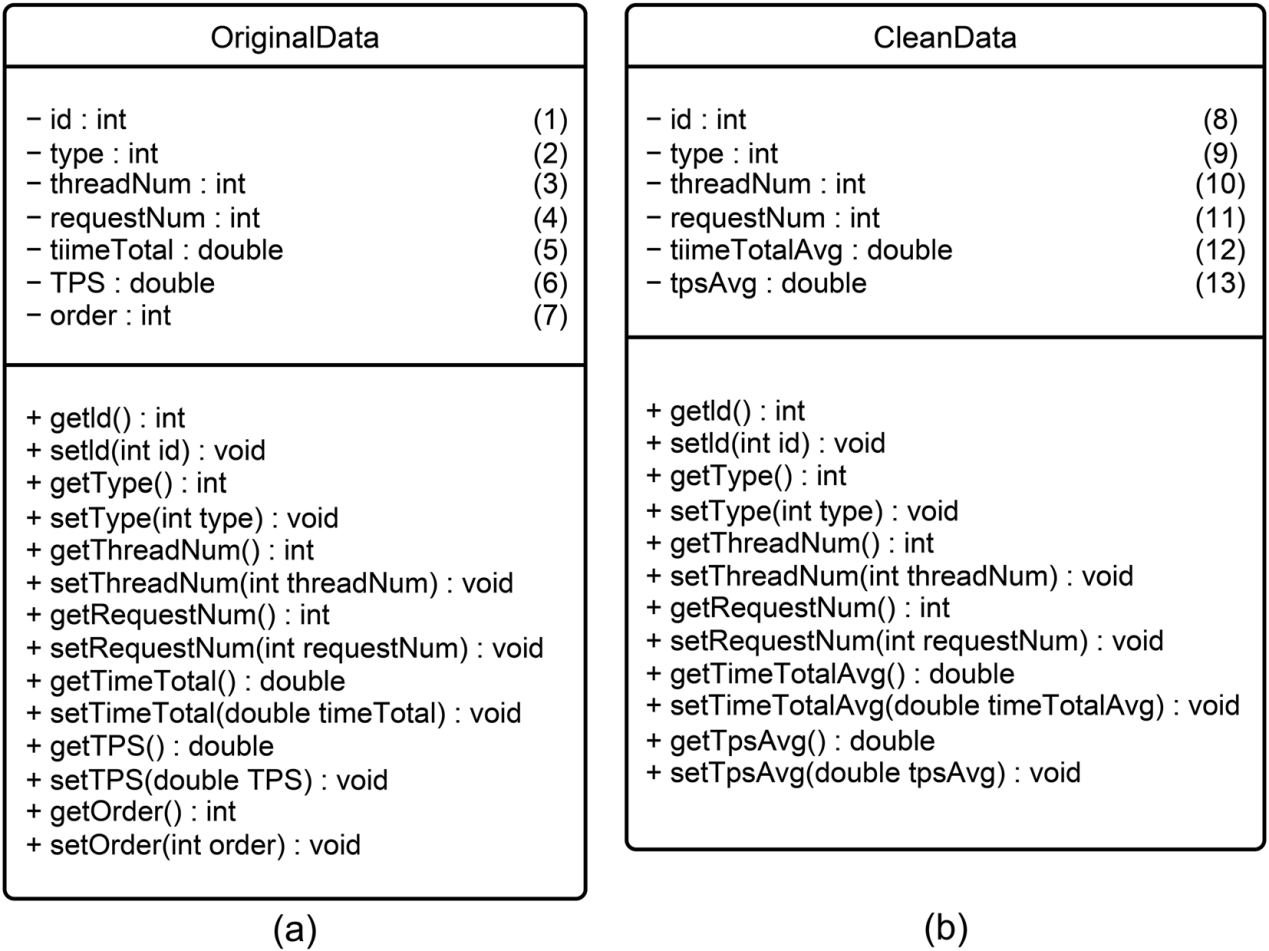
Experimental data model (**a**) original data model (**b**) cleaned data model.

First, we established an original data model and developed a data cleaning program. The original data model is shown in Fig. 7a, where (1) "id" represents the primary key of the database table with an integer data type that automatically increases with the increase in data volume; (2) "type" represents the communication technology with an integer data type, where type = 1 represents RPCX, 2 represents gRPC, and 3 represents OpenFeign; (3) "threadNum" represents the count of threads; (4) "requestNum" represents the count of requests; (5) "timeTotal" represents the total running time of the test program; (6) "TPS" represents the transactions processed per second; and (7) "order" represents the order in which the test program runs 10 times for each thread and request count combination. Therefore, the threshold of "order" is $0\le order\le 9$. The method of the original data model is to get the value and set the value for each attribute.

The original data cleaning program aims to traverse all the original data files, read the data from each file one by one, transform the data into the original data model, and map the original data model to the corresponding database fields. Using the original data model, the program inserts the original data into the database, completing the task of reading and storing the original data from the files in the database.

Through analysis of the original data model, it was found that under the same thread count and request count, each technology performed 10 rounds of operation. In each round, the first run time was significantly higher than the other 9 run times, on average 20 times higher. Taking the RPCX technology as an example, Table 2 shows a part of the original data, with field meanings as described above. Table 2 presents the run time of two rounds, each consisting of 10 runs, for thread count 10 and request counts 10 and 20, respectively. Order 0 corresponds to the first run time of each round. From Table 2, it can be seen that the first run times for request counts 10 and 20 are 944 and 947 ms, respectively, which are 20 times higher than the other nine program run times. Analysis showed that when the test program starts each round of testing, it needs to load various additional component packages, which leads to an extended first run time. Therefore, we believe that the first run time of each round of testing is not of reference value for the purpose of detecting the time performance of communication technologies in this experiment. Therefore, before analyzing the test results data, we need to clean the original result data, remove the first test program runtime data, and calculate the average of the remaining nine run times as the final experimental result of this round.

**Table 2:** Partial original data of RPCX.

id	Type	threadNum	requestNum	timeTotal (ms)	TPS	Order
1	1	10	10	944	10.593220338983052	0
2	1	10	10	39	256.4102564102564	1
3	1	10	10	41	243.9024390243902	2
4	1	10	10	27	370.3703703703704	3
5	1	10	10	32	312.5	4
6	1	10	10	40	250	5
7	1	10	10	43	232.55813953488374	6
8	1	10	10	33	303.03030303030303	7
9	1	10	10	36	277.77777777777778	8
10	1	10	10	40	250	9
11	1	10	20	947	21.11932418162619	0
12	1	10	20	42	479.1904761904762	1
13	1	10	20	33	606.0606060606061	2
14	1	10	20	32	625	3
15	1	10	20	41	487.8048780487804	4
16	1	10	20	45	444.4444444444444	5
17	1	10	20	31	645.1612903225806	6
18	1	10	20	34	588.235294117647	7
19	1	10	20	35	571.4285714285714	8
20	1	10	20	39	512.8205128205128	9

According to the data analysis strategy we have formulated, after completing the data cleaning, we established a cleaned data model, as shown in Fig. 7b, to store the cleaned data in the database. Items (8)–(11) in the cleaned data model have the same meaning as items (1)–(4) in the original data model shown in Fig. 7a. Item (12) in Fig. 7b, "timeTotalAvg", represents the average value of program runtime, with a double-precision floating point data type; and item (13), "tpsAvg", represents the average TPS, with a double-precision floating point data type. The methods for the cleaned data model are used to get and set the values for each attribute. By using the cleaned data model, we can map the cleaned data model to the new table fields in the database and store the data in the database, thus completing the data storage work after cleaning.

Due to the large volume of data, to present the performance of data processing for the three communication technologies more clearly, we sequentially expanded the data processing thread counts for the three technologies from low to high, starting from 10 threads and increasing by 100 threads, until reaching 990 threads. For each thread count, data processing for each technology starts with 10 requests and increases by 200 requests until reaching 900 requests to evaluate its performance.

**Figure 8:**
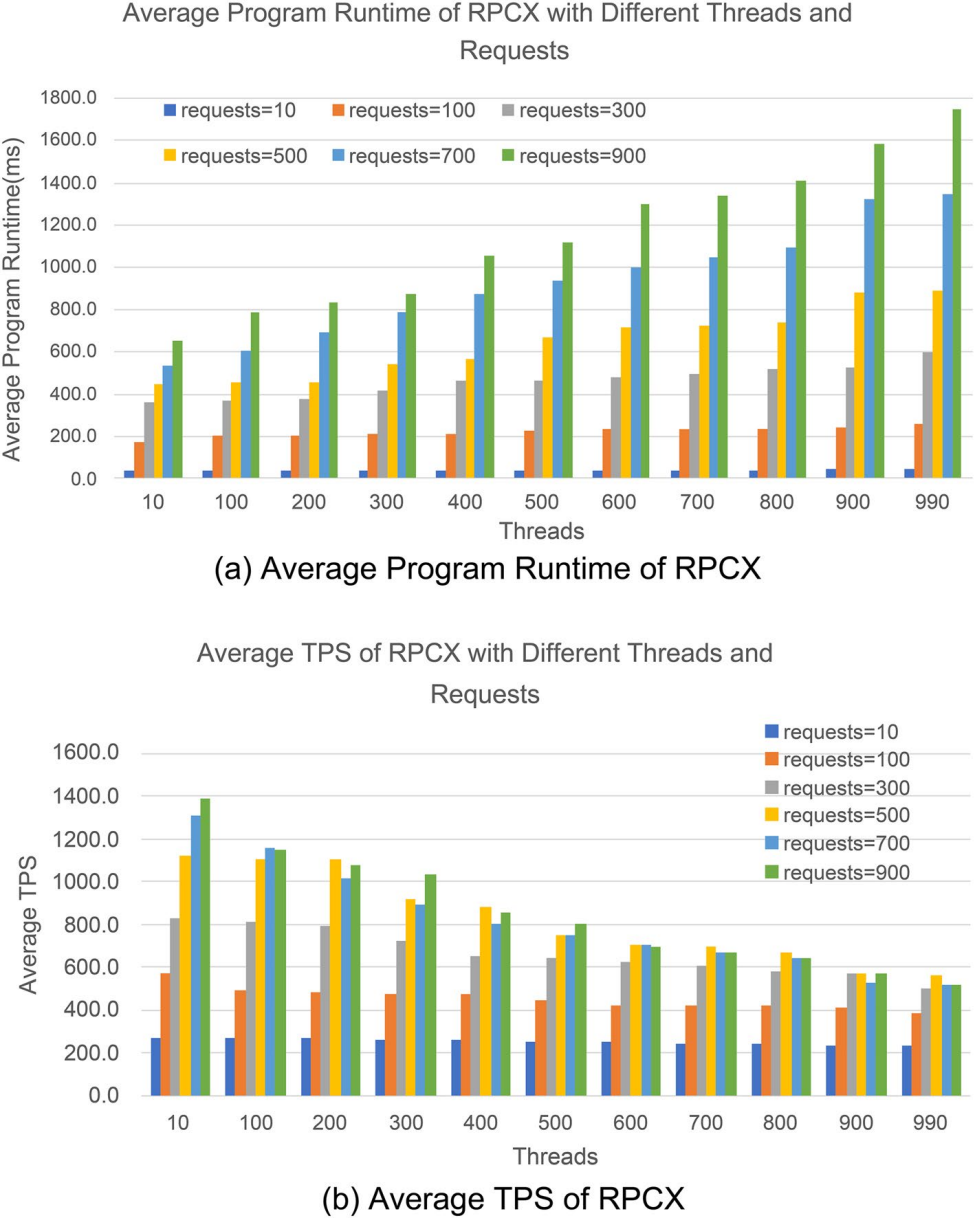
Performance of RPCX communication technology in data processing.

Figure 8 shows the performance of RPCX communication technology in terms of average program runtime and TPS, from a low thread count of 10 to a high thread count of 990. As inferred from Eqs. (1) and (2), we set the threshold values of thread and request counts, α and β, from 10 to + ∞. However, experimental results revealed that RPCX cannot demonstrate the difference in average program runtime in milliseconds when α < 10, which has no experimental significance. On the other hand, when β > 1000, the consumption of cloud server resources by RPCX communication reaches the limit, resulting in either a long execution time or the stopping of RPCX. Therefore, Fig. 8a displays the bar chart of the average program runtime of RPCX from a low thread count of 10 to a high thread count of 990. It can be observed that as the thread count increases, the program runtime of RPCX also increases, and within the same thread count, the execution time of RPCX increases as the request count increases. This distribution trend conforms to the objective law that the running time required by communication technology increases as the testing pressure increases.

Figure 8b shows the bar chart of the average TPS of RPCX from a low thread count of 10 to a high thread count of 990. As indicated by Eq. (3), when the request volume is constant, the higher the execution speed, the larger the TPS. From the figure, it can be seen that from a low thread count with a short execution time to a high thread count with a long execution time, the TPS decreases as the request volume increases. This fully conforms to the rule that communication technology has a high TPS under low response times.

**Figure 9:**
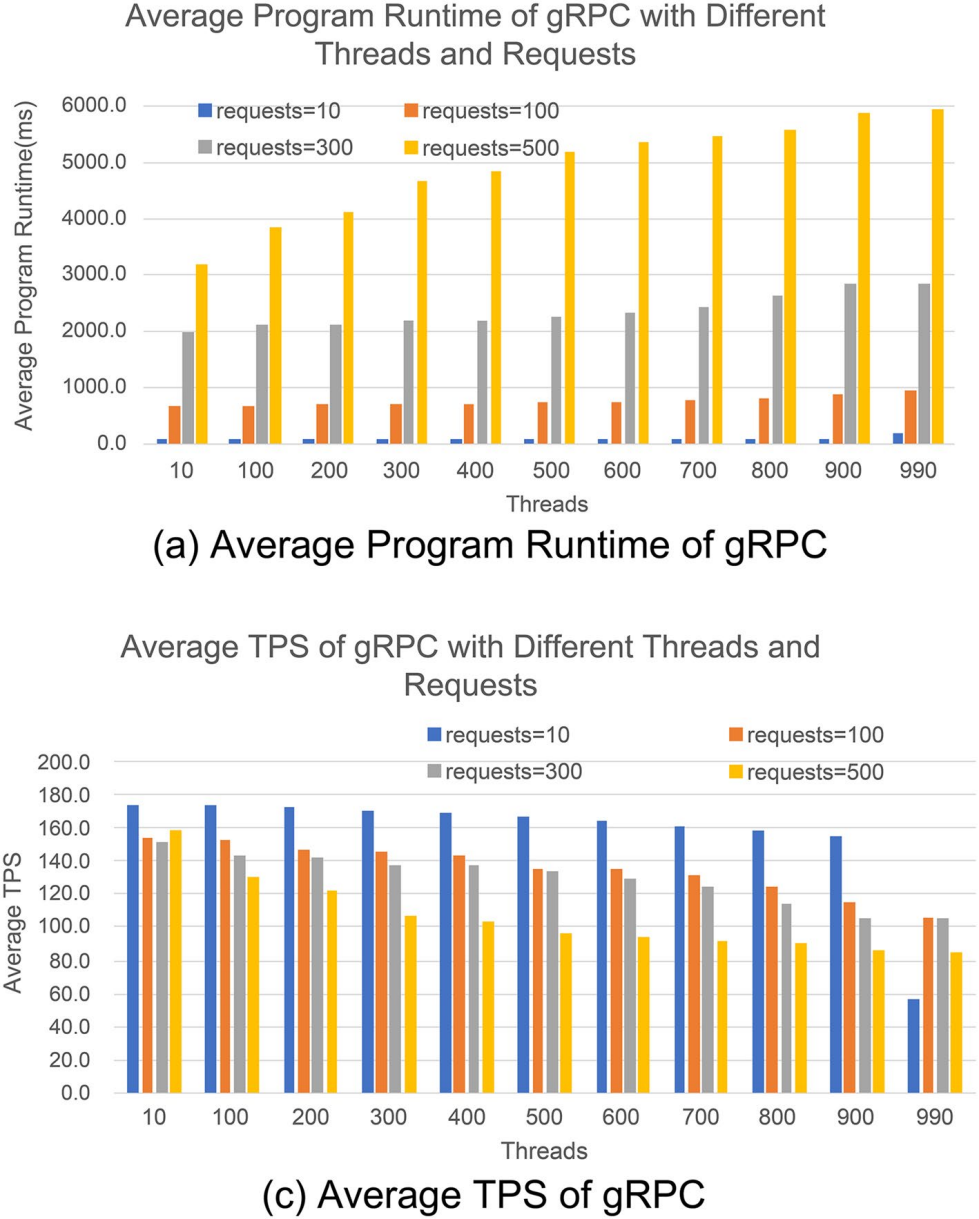
Performance of gRPC communication technology in data processing.

Figure 9a shows the bar chart of the average program running time of gRPC communication technology, starting from a low thread count of 10 with an increment of 100 to a high thread count of 990, and a request count of 10 with an increment of 200–500. According to the experimental results, the thread count threshold α = 10 and β = 990 is determined by Eq. (1), and the request count threshold α = 10 and β = 500 is determined by Eq. (2). When conducting the performance test of gRPC communication technology, the program cannot show the running time when the thread count is greater than 300 and the request count is above 500. Therefore, to display the results more clearly, the request count in the running time bar chart of gRPC is uniformly set from 10 with an increment of 200–500. Figure 9b shows the bar chart of the average TPS with a thread count ranging from 10 to a high thread count of 990 and a request count ranging from 10 to 500. Similar to RPCX, gRPC also follows the objective rule that the program running time increases and TPS decreases gradually as the performance pressure increases during the performance test.

**Figure 10:**
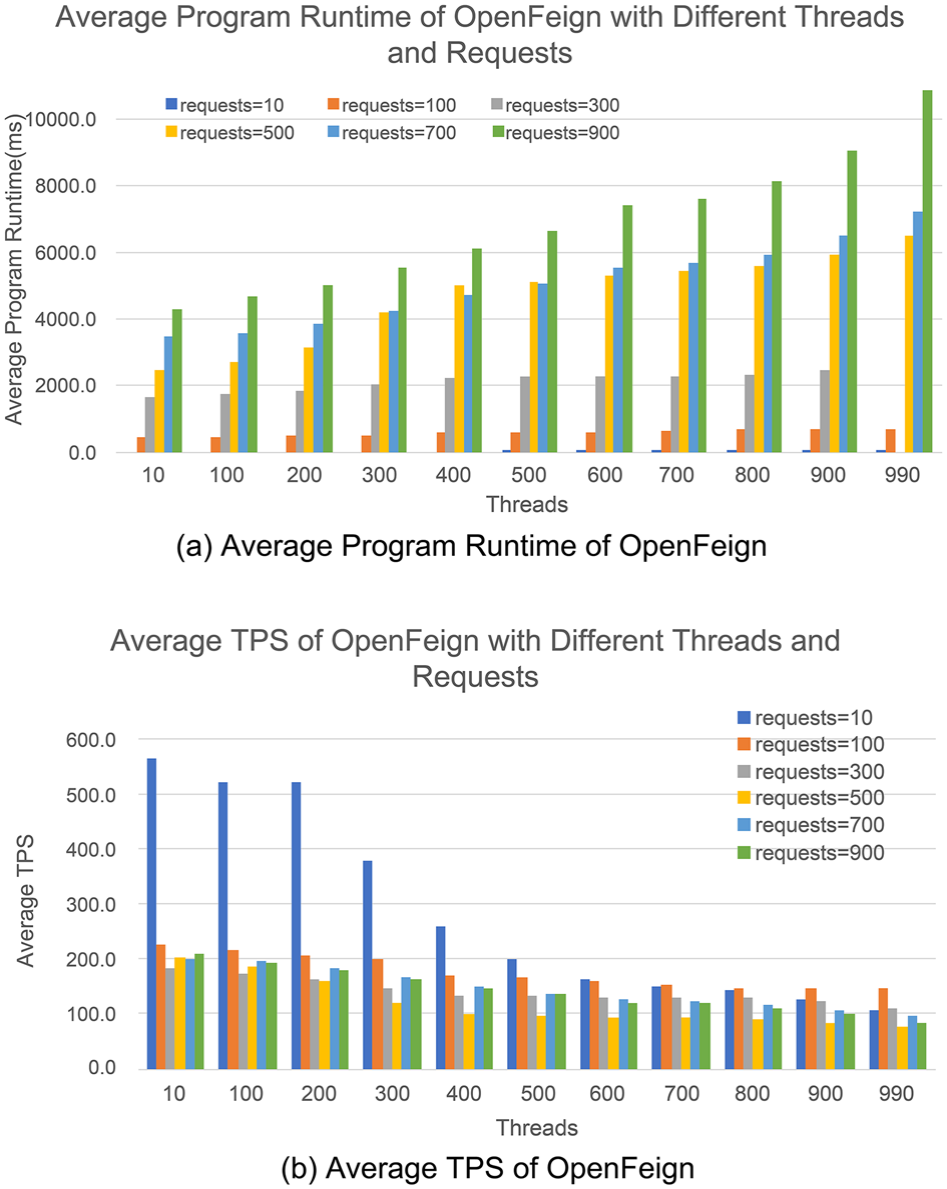
Performance of OpenFeign communication technology in data processing.

Figure 10a shows the bar chart of the average running time of programs using OpenFeign communication technology, with a low thread count of 10 and an increment of 100 up to a high thread count of 990, and a request count of 10 with an increment of 200 up to 900. The experimental results show that the thread count threshold of Eq. (1) is α = 10 and β = 990, and the request count threshold of Eq. (2) is α = 10 and β = 900. Figure 10b shows the TPS situation with thread counts ranging from 10 to 990 and request counts ranging from 10 to 900. Similar to RPCX and gRPC, OpenFeign follows the objective law that the program running time increases gradually and TPS decreases as the performance pressure continuously increases in performance stress tests.

According to the experimental data shown in Figs. 8, 9 and 10, we have compiled a comparison of the average program runtime and average TPS for the three communication technologies in Tables 3 and 4, respectively. Table 3 presents the comparison of the average program runtime for the three communication technologies from 10 threads with request counts of 100, 300, and 500, respectively, up to 990 threads. RPCX employs a caching mechanism to store target servers, remote services, and other information locally during program initialization, as described in the annotation configuration rules of the RPCX section. To further enhance the performance of RPCX, time-consuming operations such as traversing and parsing annotation classes are performed during program initialization, and the information of annotation classes is stored in the local cache pool for rapid data access during program execution. RPCX uses the non-blocking IO Netty network model and the binary data model protobuf for data transmission in the network communication model, and asynchronously transmits requests and response results, which are stored in the local cache pool in key-value format for local asynchronous calls. These design approaches greatly improve the performance of RPCX. As shown in Table 3, RPCX outperforms gRPC and OpenFeign by 55.9–88.9% in terms of time performance from low threads to high threads. Correspondingly, Table 4 presents the comparison of TPS for the three communication technologies from 10 threads with request counts of 100, 300, and 500, respectively, up to 990 threads, and RPCX outperforms gRPC and OpenFeign by 126.9–802.8% in terms of TPS from low threads to high threads.

**Table 3:** Comparison of average program runtime between RPCX and the other two technologies.

	Average program runtime														
	Requests = 100					Requests = 300					Requests = 500				
	RPCX	gRPC	DR to gRPC (%)	OpenFeign	DR to OpenFeign (%)	RPCX	gRPC	DR to gRPC (%)	OpenFeign	DR to OpenFeign (%)	RPCX	gRPC	DR to gRPC (%)	OpenFeign	DR to OpenFeign (%)
Threads															
10	175.2	648.6	73.0	442.1	60.4	362.0	1973.8	81.7	1647.9	78.0	446.9	3158.1	85.8	2449.8	81.8
100	204.6	656.2	68.8	464.2	55.9	369.6	2092.7	82.3	1730.3	78.6	452.9	3831.4	88.2	2694.8	83.2
200	206.7	679.4	69.6	482.7	57.2	379.8	2119.3	82.1	1851.4	79.5	453.4	4093.8	88.9	3125.9	85.5
300	209.0	684.7	69.4	502.1	58.4	413.0	2183.7	68.1	2036.6	79.7	541.8	4650.9	88.4	4201.3	87.1
400	211.8	695.7	69.6	585.3	63.8	462.6	2189.0	78.9	2249.1	79.4	564.1	4815.8	88.3	5020.4	88.8
500	225.2	739.1	69.5	602.9	62.6	465.9	2246.8	79.3	2271.6	79.5	668.1	5167.9	87.1	5120.8	87.0
600	235.2	743.6	68.4	621.1	62.1	477.9	2318.7	79.4	2289.8	79.1	712.0	5333.0	86.6	5325.6	86.6
700	237.4	760.7	68.8	652.0	63.6	495.4	2413.1	79.5	2301.2	78.5	719.8	5451.3	86.8	5432.2	86.7
800	238.6	799.6	70.2	675.2	64.7	517.7	2621.4	80.3	2311.0	77.6	741.8	5553.2	86.6	5574.9	86.7
900	244.0	870.4	72.0	679.4	64.1	527.8	2829.0	81.3	2452.4	78.5	879.9	5858.7	85.0	5929.2	85.2
990	260.4	942.3	72.4	686.2	62.0	597.2	2847.0	79.0	2714.8	78.0	888.2	5923.1	85.0	6522.1	86.4

*DR* decrease rate of average program runtime.

**Table 4:** Comparison of average TPS between RPCX and the OTHER TWO TECHNOLOGIES.

	Average PTPS														
	Requests = 100					Requests = 300					Requests = 500				
	RPCX	gRPC	IR to gRPC (%)	OpenFeign	IR to OpenFeign (%)	RPCX	gRPC	IR to gRPC (%)	OpenFeign	IR to OpenFeign (%)	RPCX	gRPC	IR to gRPC (%)	OpenFeign	IR to OpenFeign (%)
Threads															
10	570.7	154.2	270.2	226.2	152.3	828.7	152.0	445.2	182.1	355.2	1118.8	158.3	606.7	204.1	448.2
100	488.9	152.4	220.8	215.4	126.9	811.8	143.4	466.3	173.4	368.2	1104.0	130.5	746.0	185.5	495.0
200	483.9	147.2	228.8	207.2	133.6	789.9	141.6	458.0	162.0	387.5	1102.7	122.1	802.8	160.0	589.4
300	478.5	146.1	227.6	199.2	140.2	726.4	137.4	428.7	147.3	393.1	922.9	107.5	758.4	119.0	675.5
400	472.2	143.7	228.5	170.8	176.4	648.6	137.0	373.2	133.4	386.2	886.4	103.8	753.7	99.6	790.0
500	444.0	135.3	228.2	165.9	167.7	643.9	133.5	382.3	132.1	387.6	748.4	96.8	673.5	97.6	666.5
600	425.1	134.5	216.1	161.0	164.0	627.8	129.4	385.2	131.0	379.1	702.2	93.8	649.0	93.9	648.0
700	421.2	131.5	220.4	153.4	174.6	605.5	124.3	387.1	130.4	364.5	694.7	91.7	657.4	92.0	654.7
800	419.2	125.1	235.2	148.1	183.0	579.5	114.4	406.4	129.8	346.4	674.1	90.0	648.6	89.7	651.6
900	409.8	114.9	256.7	147.2	178.5	568.4	106.0	436.0	122.3	364.7	568.3	85.3	565.9	84.3	573.9
990	384.0	106.1	261.8	145.7	163.5	502.3	105.4	376.7	110.5	354.6	562.9	84.4	566.9	76.7	634.3

*IR* improvement rate of average TPS.

## Conclusions

We designed a new microservice service communication technology called RPCX and compared it with gRPC and OpenFeign in terms of stress performance. According to the results, RPCX exhibits good service communication time and TPS performance. The novel method proposed in this study can improve the performance of communication technology in the field of service communication in the microservice architecture and can help future researchers further improve the communication performance, ease of use of microservices and promote development in the field of microservices IPC technology. In the future, experiments should be extended to multiple cloud hosts and across hosts, and more complex experimental plans should be developed.

## Data Availability

The datasets generated during and/or analyzed during the current study are available from the corresponding author on reasonable request.
